# [Corrigendum] Small molecule CDS-3078 induces G^2^/M phase arrest and mitochondria-mediated apoptosis in HeLa cells

**DOI:** 10.3892/etm.2025.12938

**Published:** 2025-08-06

**Authors:** Yuanxin Zhang, Pengcheng Li, Jiamin Rong, Yakun Ge, Chenming Hu, Xu Bai, Wei Shi

Exp Ther Med 20:284, 2020; DOI: 10.3892/etm.2020.9414

Subsequently to the publication of the above article, an interested reader drew to the Editor’s attention that there may have been an issue of non-specificity associated with the choice of the anti-Bax antibody (sc-7480) in light of a publication that appeared in the journal *Cell Death & Disease* written by Entrop and colleagues on this issue; furthermore, it was noted that, for the western blots shown in Fig. 3A on p. 6, there appeared to have been a duplication of data that concerned the Procaspase 3/Cleavage caspase 3 and Procaspase 9/Cleavage caspase 9 experiments.

First, the authors acknowledged the possibility of false-positive results using the anti-Bax antibody, although they did not identify any regulatory effects of the small-molecule CDS-3078 using this antibody in their experiments; furthermore, the regulatory effects of CDS-3078 were explored on the basis of its influence on the proteins Bcl-2, Bak and cytochrome *c*, which was sufficient to demonstrate its ability to induce apoptosis. Secondly, they realized that Fig. 3 had inadvertently been assembled incorrectly; however, the authors had retained their orginal data, and were able to provide a revised version of Fig. 3, now featuring the correct data for the Procaspase 9/Cleavage caspase 9 experiment. The revised version of Fig. 3 is shown on the next page. Note that the error made in the assembly of Fig. 3 did not have a major impact on either the overall results or on the conclusions reported in this study. All the authors agree with the publication of this corrigendum, and are grateful to the Editor of *Experimental and Therapeutic Medicine* for granting them the opportunity to publish this; furthermore, they apologize to the readership for any inconvenience caused.

## Figures and Tables

**Figure 3:**
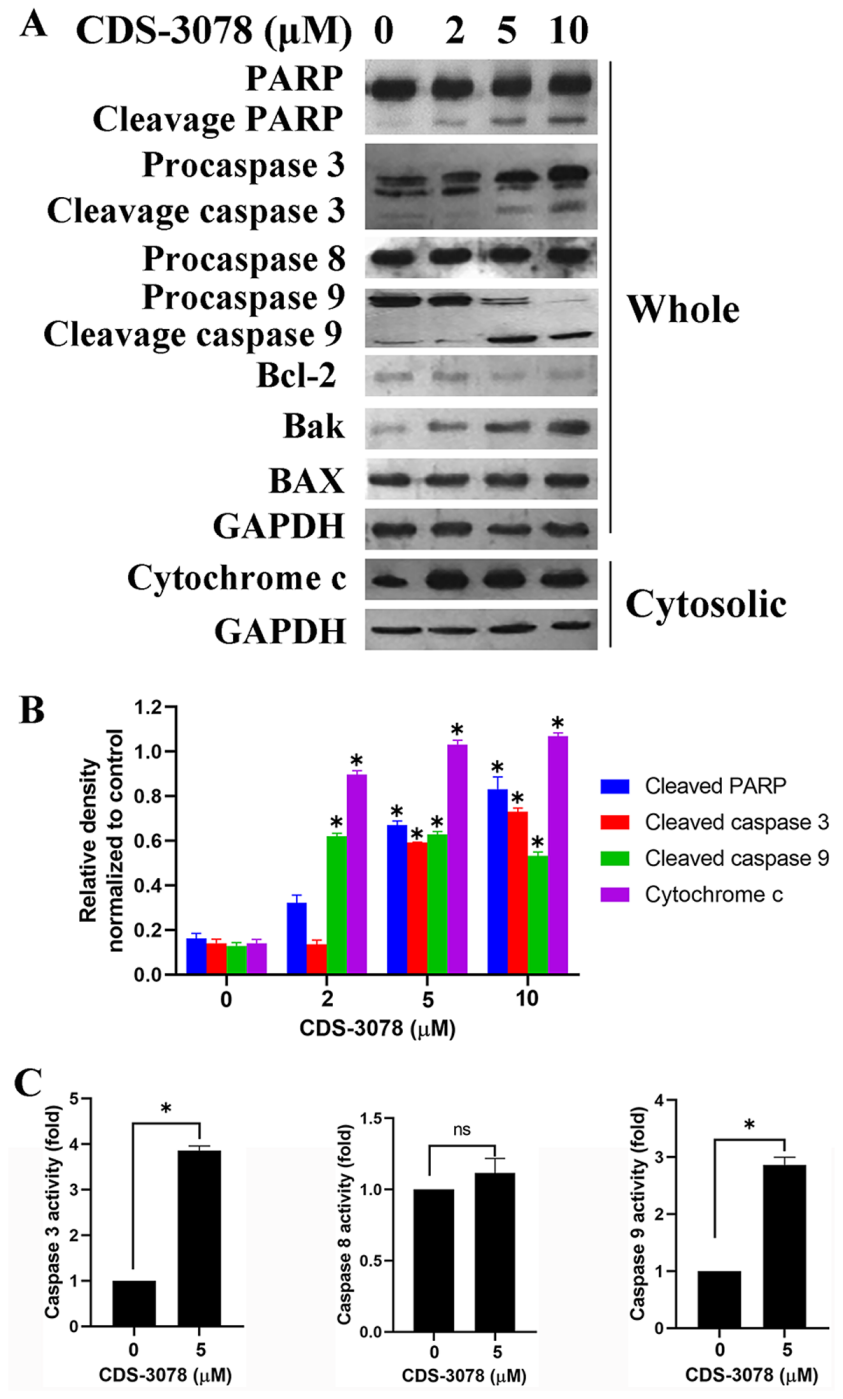
CDS-3078 promotes mitochondria-mediated dysfunction and regulates the protein expression of Bcl-2 family. (A) Cells were treated with various concentrations of CDS-3078 (2, 5 and 10 µM) for 24 h, cytosolic extracts and whole-cell lysates were detected by immunoblotting to examine the expression levels of cytochrome c, Bcl-2, BAK, BAX, PARP and caspase-3/8/9. (B) The relative density was determined using grayscale analysis on ImageJ v 1.8.0_112 (National Institutes of Health), normalized to GAPDH. (C) Cells were treated with 5 µM CDS-3078 for 12 h. Equal amounts of cell lysates were analyzed for caspase-3, caspase-8 and caspase-9 activity using Ac-DEVD-AFC, Ac-LETD-AFC, Ac-LEHD-AFC as substrates, respectively. DMSO treatment was used as the control. The concentration of the fluorescent products released were then measured. Results represent the mean ± SD of three independent repeats. ^*^P<0.05 vs. 0 µM CDS-3078 control. CDS-3078, 2-[1-(4-(Benzyloxy)phenyl)-3-oxoisoindolin-2-yl)-2-(4-methoxyphenyl)] acetic acid; PARP, poly ADP-ribose polymerase.

